# P-2291. Low-level CMV Viremia After Solid Organ Transplant: Should We Treat?

**DOI:** 10.1093/ofid/ofae631.2444

**Published:** 2025-01-29

**Authors:** Rohan Goyal, Daniel A Burack, Erika Orner, Clara Tow, Sammar R Alsunaid, Yogita Rochlani, Periklis Kyriazis, Rachel Bartash, Margaret E McCort

**Affiliations:** Montefiore Medical Center, Melville, New York; Montefiore Medical Center, Melville, New York; Montefiore Medical Center, Melville, New York; Montefiore Medical Center, Melville, New York; Montefiore Medical Center/Albert Einstein College of Medicine, New York, New York; Montefiore Medical Center, Albert Einstein College of Medicine, Bronx, New York; Albert Einstein School of Medicine, New York, New York; Montefiore Medical Center, Melville, New York; Montefiore Medical Center / Albert Einstein College of Medicine, Bronx, New York

## Abstract

**Background:**

Cytomegalovirus (CMV) disease is known to affect morbidity and mortality after solid organ transplant (SOT). However, less is known concerning low-level CMV viremia (llCMV) (< 1000 IU/ml). Having llCMV and the decision to treat llCMV with antivirals may be associated with changes in long-term graft outcomes and mortality in SOT. We aimed to review patient-level outcomes of llCMV after SOT in our large urban academic medical center.

**Figure:**
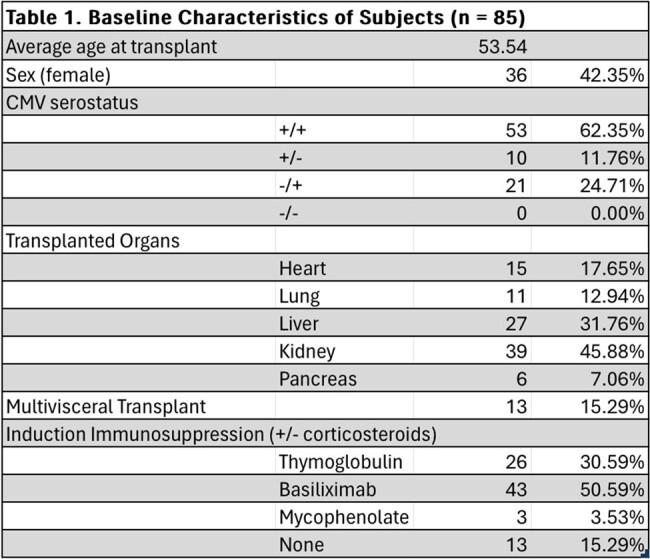

**Methods:**

We performed a retrospective review of patients from Montefiore Medical Center who underwent heart, liver, lung, kidney, pancreas, or multi-visceral transplant between 2020-2021 and developed llCMV within 2 years of SOT. Chart review was performed to characterize how llCMV was managed and outcomes such as graft failure, graft rejection, rehospitalization, concomitant infections, and death relative to llCMV detection.

**Figure 1.**
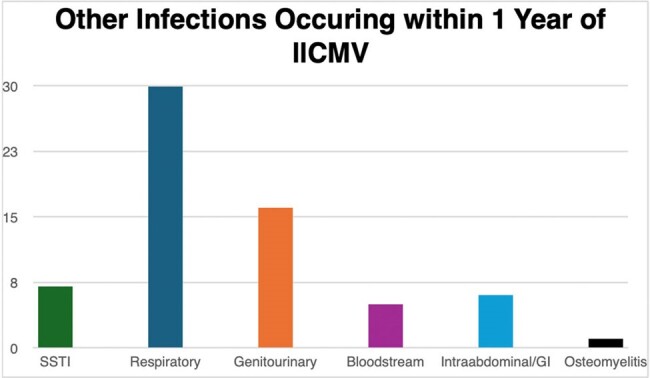
Distribution of other infections affecting solid organ transplant recipients within 1 year of low level CMV viremia.

**Results:**

Eighty-five adult SOT recipients who had CMV viral loads measured between 50 and 1000 IU/mL within two years of transplant were identified. See Table 1 for recipient details. The average time from transplant to llCMV was 272 days. Seventeen (20%) patients were receiving antiviral prophylaxis at the time of llCMV, while post-transplant CMV prophylaxis had been discontinued in 66 patients (78%) for an average of 149 days prior to llCMV. 64% (54/85) were started on antivirals or had an antiviral dose increase in response to llCMV, which was continued for an average of 109 days. Within a year of llCMV, 42 (49%) of SOT recipients were re-hospitalized. Forty-two (49%) of patients experienced non-CMV infections within 1 year of llCMV detection; see Figure 1. Graft rejection occurred in 25 (29%) of patients with llCMV, and 13 (15%) experienced graft failure. Mortality within 1 year of llCMV was 8% (7/85).

**Conclusion:**

llCMV is likely a reflection of overall immune status and is often temporally associated with rehospitalization and concomitant infections. Graft rejection and death are uncommon in adult SOT recipients with llCMV within 2 years of transplant. Although antiviral use was common in response to llCMV, the benefit of this is unknown and comparison to outcomes of SOT recipients without llCMV or those not receiving therapy for llCMV is needed to better determine when treatment is needed.

**Disclosures:**

All Authors: No reported disclosures

